# Prospective validation of microRNA signatures for detecting pancreatic malignant transformation in endoscopic-ultrasound guided fine-needle aspiration biopsies

**DOI:** 10.18632/oncotarget.8699

**Published:** 2016-04-12

**Authors:** Adam E. Frampton, Jonathan Krell, Mireia Mato Prado, Tamara M.H. Gall, Nima Abbassi-Ghadi, Giovanna Del Vecchio Blanco, Niccola Funel, Elisa Giovannetti, Leandro Castellano, Mohamed Basyouny, Nagy A. Habib, Harry Kaltsidis, Panagiotis Vlavianos, Justin Stebbing, Long R. Jiao

**Affiliations:** ^1^ HPB Surgical Unit, Department of Surgery and Cancer, Imperial College, Hammersmith Hospital, London, UK; ^2^ Division of Cancer, Department of Surgery and Cancer, Imperial College, Hammersmith Hospital, London, UK; ^3^ Academic Surgical Unit, Department of Surgery and Cancer, Imperial College, St. Mary's Hospital, London, UK; ^4^ Department of Systems Medicine, Gastroenterology Unit, University Tor Vergata, Rome, Italy; ^5^ Cancer Pharmacology Lab, AIRC Start-Up Unit, Department of Translational Research and New Technologies in Medicine and Surgery, University of Pisa, Pisa, Italy; ^6^ CNR-Nano, Institute of Nanoscience and Nanotechnology, Pisa, Italy; ^7^ Department of Medical Oncology, VU University Medical Center, Amsterdam, Netherlands; ^8^ Department of Gastroenterology, Imperial College Healthcare NHS Trust, Hammersmith Hospital, London, UK

**Keywords:** microRNA, endoscopic ultrasound (EUS), fine-needle aspiration (FNA), pancreatic ductal adenocarcinoma, pancreatic cyst

## Abstract

**Background:**

Pancreatic ductal adenocarcinoma (PDAC) is a lethal disease. Novel biomarkers are required to aid treatment decisions and improve patient outcomes. MicroRNAs (miRNAs) are potentially ideal diagnostic biomarkers, as they are stable molecules, and tumour and tissue specific.

**Results:**

Logistic regression analysis revealed an endoscopic-ultrasound fine-needle aspiration (EUS-FNA) 2-miRNA classifier (miR-21 + miR-155) capable of distinguishing benign from malignant pancreatic lesions with a sensitivity of 81.5% and a specificity of 85.7% (AUC 0.930). Validation FNA cohorts confirmed both miRNAs were overexpressed in malignant disease, while circulating miRNAs performed poorly.

**Methods:**

Fifty-five patients with a suspicious pancreatic lesion on cross-sectional imaging were evaluated by EUS-FNA. At echo-endoscopy, the first part of the FNA was sent for cytological assessment and the second part was used for total RNA extraction. Candidate miRNAs were selected after careful review of the literature and expression was quantified by qRT-PCR. Validation was performed on an independent cohort of EUS-FNAs, as well as formalin-fixed paraffin embedded (FFPE) and plasma samples.

**Conclusions:**

We provide further evidence for using miRNAs as diagnostic biomarkers for pancreatic malignancy. We demonstrate the feasibility of using fresh EUS-FNAs to establish miRNA-based signatures unique to pancreatic malignant transformation and the potential to enhance risk stratification and selection for surgery.

## INTRODUCTION

Over the last decade, we have seen a steady rise in the incidence of pancreatic cysts, estimated to be present in ~2.4% of the population [1, 2]. This is largely due to an increase in the use of cross-sectional imaging investigations undertaken for other symptoms, and an improvement in high-definition radiological techniques. There are a number of different pancreatic cysts and lesions which demand varying treatment protocols. For example, benign and inflammatory pancreatic cysts with no malignant potential, including pseudocysts, inflammatory masses, and serous cystadenomas (SCA) [3], have either no risk or a very low risk of malignant change, and asymptomatic lesions can be managed conservatively with long-term follow-up. Whereas mucinous lesions, like mucinous cystic neoplasms (MCN) and intraductal papillary mucinous neoplasms (IPMN), are considered pre-malignant lesions and require more careful consideration. By correctly diagnosing these lesions as early as possible, we can ensure that the appropriate management plan is initiated and patients do not undergo unnecessary major surgery or those requiring resection do not have any delay to surgery.

Computer tomography (CT) and magnetic resonance imaging (MRI) are unable to accurately distinguish between benign and malignant pancreatic cystic lesions [4] and therefore the majority of patients undergo an endoscopic ultrasound scan (EUS) and fine needle aspiration (FNA) for cytology and cystic fluid analysis. EUS can differentiate between benign, pre-malignant and malignant lesions with an accuracy ranging from 40 to 93% [5–7]. FNA can add to the diagnosis by allowing cytological analysis of the fluid. Despite having a high specificity for malignancy, fluid from cystic lesions tends to be paucicellular, thus making the sensitivity of this test in the order of 35–50% [8, 9]. In addition, cyst fluid carcinoembryonic antigen (CEA) is routinely measured, as this is currently the best biomarker for identifying malignant change. Nevertheless, it is estimated that these investigative efforts still lead to inaccuracies in diagnosis resulting in 20% of patients being incorrectly identified as having a pre-malignant or malignant lesion and undergoing surgical resection, and 7% being falsely labelled as having benign lesions [10].

Consequently, there is an urgent need to establish an alternative EUS-FNA/cystic fluid biomarker with improved diagnostic accuracy. MicroRNAs (miRNA) are single-stranded small RNA molecules involved in the post-transcriptional regulation of gene expression and are crucial in tumourigenesis. Indeed, miRNAs have been shown to be important regulators of apoptosis, proliferation, invasion and metastasis in various human cancers [11]. The differential expression of various miRNAs has also been implicated in PDAC [12], with some miRNA expression patterns being associated with poor survival outcomes [13]. Furthermore, studies have demonstrated that the inhibition of selected oncogenic miRNAs can reduce PDAC tumour growth *in vivo* by de-repressing a network of tumour suppressors, indicating therapeutic potential [13]. Other studies have shown that miRNA profiles can also differentiate between benign and malignant cystic lesions [14–19]. In addition, a few small studies have shown that the detection of miRNAs in FNA samples taken at EUS is feasible and may increase the sensitivity and specificity of detecting malignant change in cystic lesions of the pancreas [17, 20–23].

The aim of this study was to prospectively assess the role of cancer-specific miRNAs in the detection of pancreatic malignancy from EUS-FNAs.

## RESULTS

### Initial pancreatic EUS-FNA samples

In the initial cohort, 55 EUS-FNAs were obtained from suspicious pancreatic lesions and had miRNA expression analysis performed. The demographics and clinical characteristics for the patients included in the study are summarised in Table 1. Mean age was 64.6 ± 13.8 years; 50.9% were female and patients with carcinoma present were more likely to be symptomatic and have a more solid lesion. According to echo-endoscopy, of these 55 pancreatic lesions, 61.8% (*n* = 34) were classified as solid or having solid elements, whilst 38.2% (*n* = 21) were entirely cystic. Patients with carcinomas had more needle passes, probably due to identification of a solid component at echo-endoscopy, and also higher RNA yields. Of the 55 patients having EUS-FNA, 29% (*n* = 16) went on to have surgical resection: 2 patients with chronic pancreatitis (CP); 7 IPMNs and 7 PDACs.

**Table 1:** Demographic and clinical description of initial EUS-FNA study cohort

	Benign/Inflammatory/Pseudocyst (*n* = 18)	Mucinous (*n* = 10)	CEI (*n* = 9)	PDAC (*n* = 18)	*P*
**Age (years)**	56.7 ± 17.5	64.4 ± 11.5	73.9 ± 4.2	68.0 ± 9.6	**0.008¥^†^**
**Female gender (%)**	11 (61.1%)	6 (60%)	6 (66.7%)	5 (27.8%)	0.120^☼^
**Symptoms**	8 (44.4%)	4 (40%)	6 (66.7%)	15 (83.3%)	0.052^☼^
**Size (mm)**	21.8 ± 13.5	17.2 ± 4.69	22.7 ± 13.4	28.4 ± 9.7	0.081^¥^
**Solid or cystic**	7/11	4/6	5/4	18/0	**0.001^☼^**
**No. of fine needle passes**	1.1 ± 0.4	1.2 ± 0.4	1.7 ± 0.9	3.2 ± 1.4	**< 0.0001^¥^^‡^**
**RNA yield (ng/μl)**	50.0 ± 60.9	44.5 ± 43.0	248.9 ± 286.3	117.3 ± 109.5	**0.004^¥^^§^**

☼ Chi-square test.

¥ 1-way ANOVA.

† Benign/Inflammatory/Pseudocyst vs. CEI or PDAC.

‡ PDAC vs. Benign/Inflammatory/Pseudocyst, Mucinous or CEI.

§ CEI vs.

Benign/Inflammatory/Pseudocyst or Mucinous. PDAC, pancreatic ductal adenocarcinoma; CEI, carcinoma-ex-IPMN (i.e. invasive IPMN).

Four groups of patients underwent EUS-FNA for suspicious pancreatic lesions, including those with: benign/inflammatory/pseudocysts (*n* = 18; comprising of 4 SCAs and 14 inflammatory/pseudocysts); mucinous cysts (*n* = 10; 5 main duct (MD)-IPMNs, 2 branch duct (BD)-IPMNs and 3 MCNs); invasive IPMN or carcinoma-ex-IPMN (CEI; *n* = 9) and PDAC (*n* = 18). At cytological assessment for malignancy, the frequency of indeterminate results (i.e. insufficient, C1; atypical, C3; or suspicious, C4) was 36.4% (20 of 55; Table 2). Therefore, for the remaining 35 cases, EUS-FNA cytology alone had a sensitivity of 94%, and a specificity of 100%, using stringent criteria (i.e. C2 indicates benign, and C5 indicates malignant). Using less stringent criteria (i.e. given clinical context and results from other investigations, the C2 and C3 can be combined to define “benign disease”; whilst C3 and C4 can be combined to define “malignant disease”), EUS-FNA cytology had a sensitivity of 94%; however specificity was reduced to 75%.

**Table 2:** Comparison of EUS-FNA cytology results with reference standards for initial study cohort

	Benign/Inflammatory/Pseudocyst (*n* = 18; %)	Mucinous (*n* = 10; %)	CEI (*n* = 9; %)	PDAC (*n* = 18; %)	Total (%)
**Insufficient (C1^†^)**	4 (22.2)	1 (10)	1 (11.1)	1 (5.6)	7 (12.7)
**Benign (C2)**	13 (72.2)	2 (20)	1 (11.1)	0 (0)	16 (29.1)
**Atypical cells (C3^†^)**	1 (5.6)	7 (70)	3 (33.3)	0 (0)	11 (20)
**Suspicious (C4^†^)**	0 (0)	0 (0)	0 (0)	2 (11.1)	2 (3.6)
**Malignant (C5)**	0 (0)	0 (0)	4 (44.4)	15 (83.3)	19 (34.5)

Reference Standard is taken to be the combination of results and MDT opinion or surgical histology. CEI, carcinoma-ex-IPMN; PDAC, pancreatic ductal adenocarcinoma.

† C1, C3 and C4 were considered indeterminate results.

### MiRNAs can be used to detect pancreatic malignancy in EUS-FNAs

**Figure 1:**
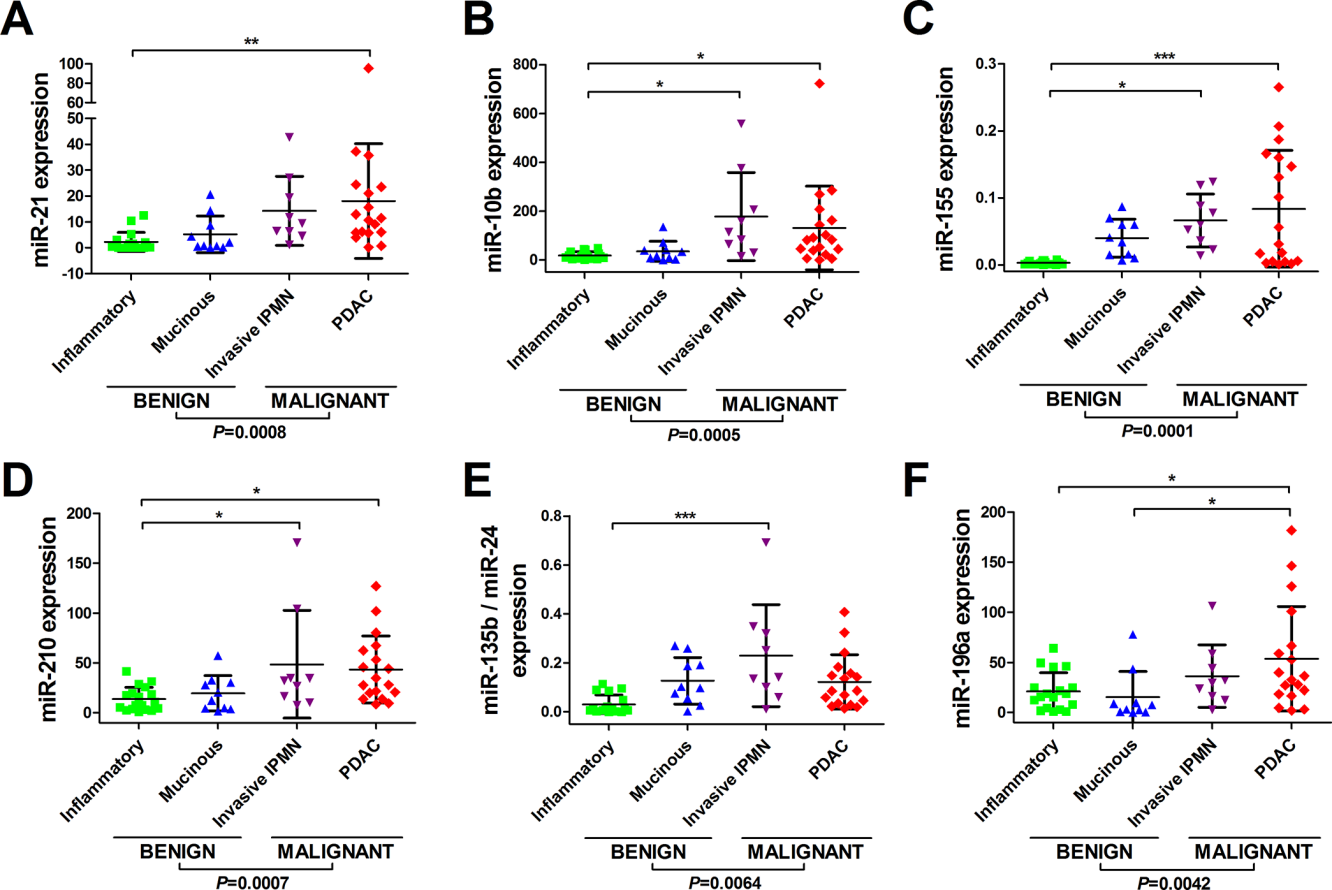
Differential miRNA expression can be detected between benign, premalignant, and malignant pancreatic lesions in EUS-FNAs Displayed are the expression levels across tissue-types for (**A**) miR-21, (**B**) miR-10b, (**C**) miR-155, (**D**) miR-210, (**E**) miR-135/miR-24 and (**F**) miR-196a. Total RNA was isolated from EUS-FNAs and quantitative real-time reverse-transcription PCR (qRT-PCR) was used to quantify miRNA expression levels. Small nuclear RNA U6 was used as an endogenous control. Scatterplots are shown for each miRNA and the horizontal lines represent the mean expression level and standard deviation. Four groups of pancreatic lesion had EUS-FNA: benign/inflammatory/pseudocysts (*n* = 18); mucinous cysts (*n* = 10); invasive IPMNs (*n* = 9) and PDAC (*n* = 18). One-way analysis of variance (ANOVA) was used to compare miRNA levels between tissue-types, followed by Tukey's multiple comparison test (***P < 0.001; **P < 0.01; *P < 0.050). Two-tailed Student's *t*-test was used to compare benign vs. malignant groups.

When compared against the final diagnoses, five individual miRNAs (i.e. miR-21, miR-155, miR-210, miR-196a and miR-10b) and also the 2-miRNA classifier, miR-135b/24, were found to be significantly up-regulated (all *P* < 0.01) in malignant pancreatic lesions (i.e. invasive IPMNs and PDAC), compared to benign lesions (i.e. SCAs, pseudocysts, MCNs and IPMN adenomas; Figure 1A–1F). However, none were able to differentiate between non-mucinous and mucinous cysts (Figure 1A–1F). Expression levels for miR-217 were too low to be determined (data not shown) and therefore this miRNA was not considered further. Thus, it was not possible to calculate the 2-miRNA classifier miR-196a/217 as we had planned.

**Figure 2:**
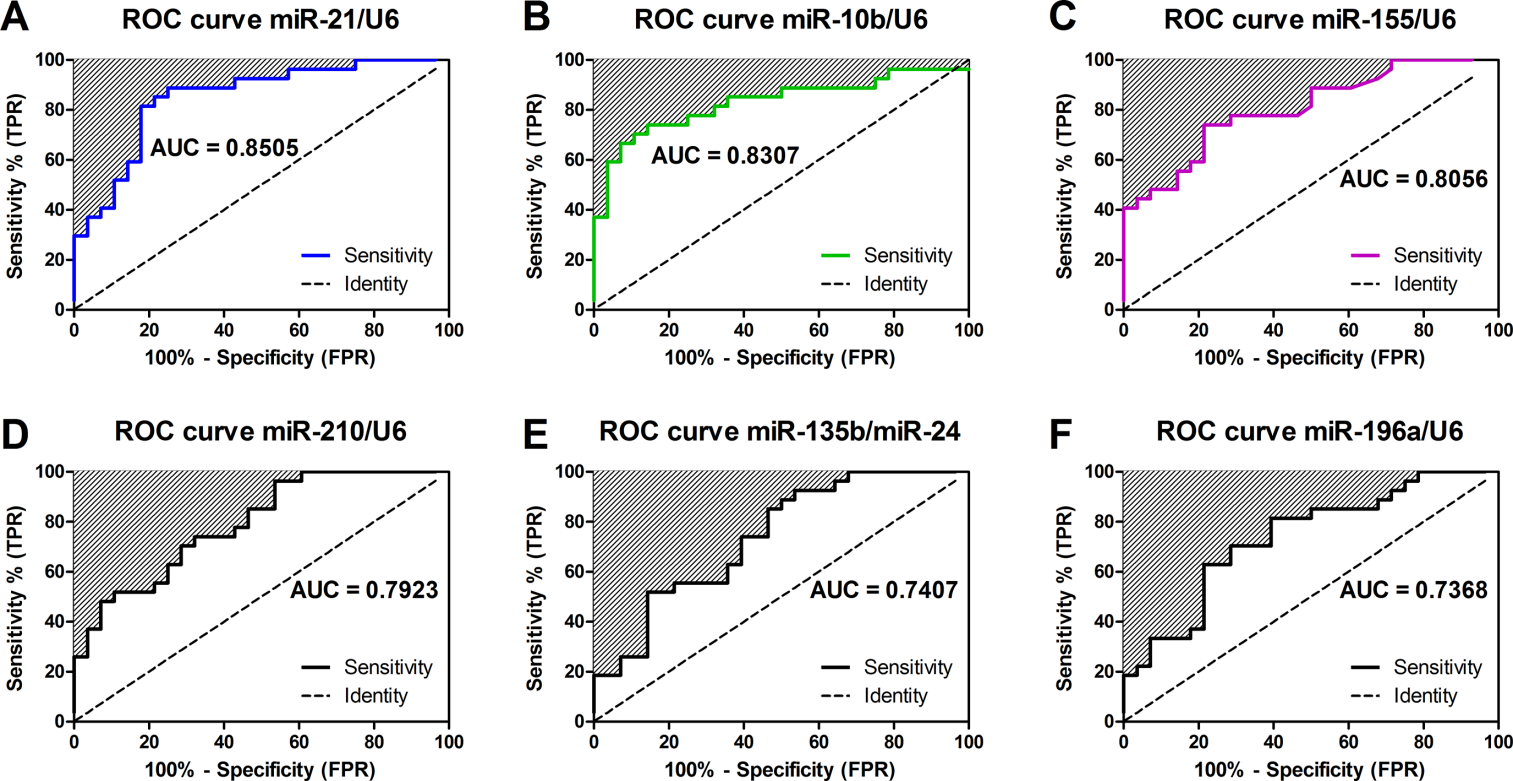
Performance of miRNAs for detecting pancreatic malignancy in EUS-FNAs Displayed are the receiver operating characteristic (ROC) curves and Area Under the Curves (AUC) for (**A**) miR-21, (**B**) miR-10b, (**C**) miR-155, (**D**) miR-210, (**E**) miR-135/miR-24 and (**F**) miR-196a. AUC measures discrimination, that is, the ability of the test to correctly classify those with and without malignancy. ROC curves show the true positive rate on vertical axis and false positive rate on horizontal axis.

Next, we assessed the performance of these miRNA at discriminating benign from malignant disease by ROC curve analysis and Area Under Curve (AUC) measurement. We found that 4 miRNAs (Figure 2) had an AUC > 0.75, indicating that they may be clinically useful biomarkers. These included miR-21 (AUC 0.851, 95% CI 0.748–0.953; Figure 2A), miR-10b (AUC 0.831, 95% CI 0.716–0.945; Figure 2B), miR-155 (AUC 0.806, 95% CI 0.692–0.919; Figure 2C) and miR-210 (AUC 0.792, 95% CI 0.677–0.908; Figure 2D). The miR-135b/24 classifier (Figure 2E) and miR-196a (Figure 2F) had AUC < 0.75 and therefore poor discriminatory power.

### A miR-21 and miR-155 classifier has the best discriminatory power for pancreatic malignancy in EUS-FNAs

**Figure 3:**
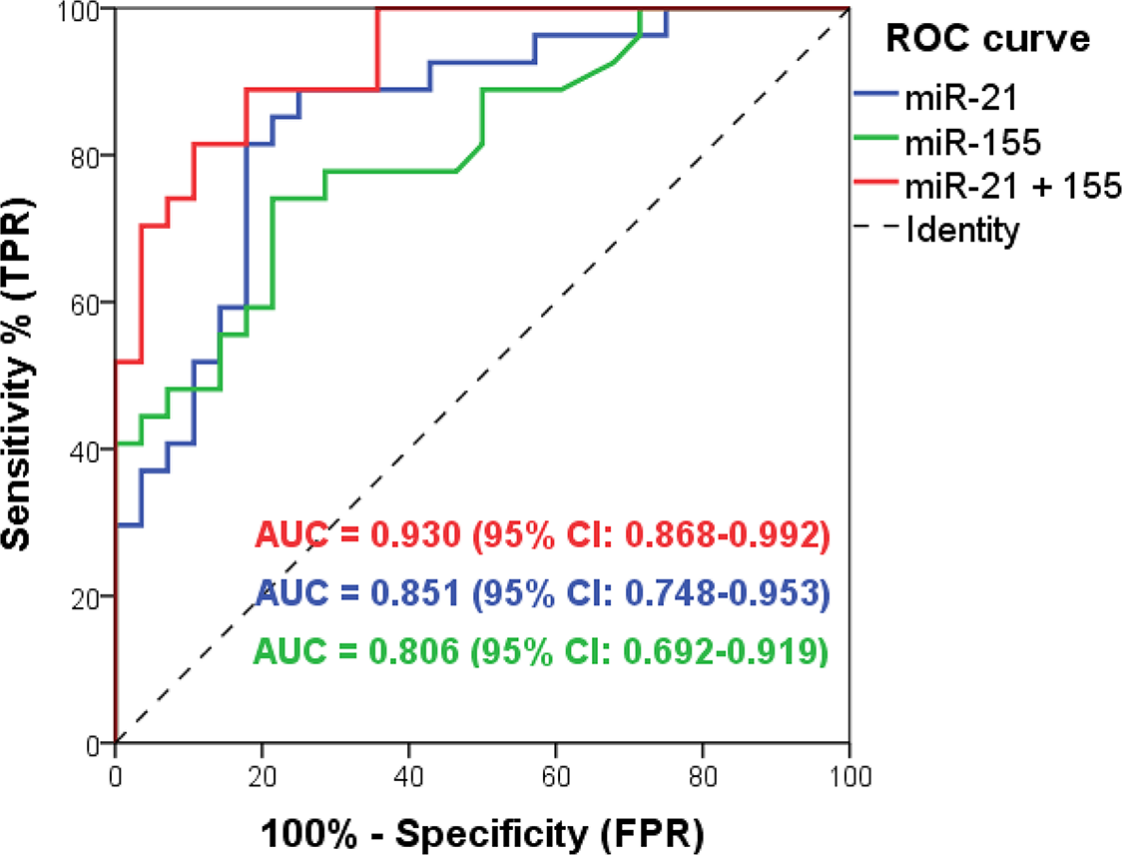
The discriminative ability of the EUS-FNA miR-21 and miR-155 classifier between malignant and benign pancreatic disease by Receiver Operating Characteristic (ROC) curve analysis MiR-21 + miR-155 combined (red line; AUC 0.930, sensitivity 81.5%, specificity 85.7%); miR-21 (blue line; AUC 0.851); and miR-155 (green line; AUC 0.806).

Using multivariate binary logistic regression, we identified which of the miRNAs were important biomarkers for pancreatic carcinoma in the initial EUS-FNA dataset. This showed that both miR-21 and miR-155 were strong independent predictors of pancreatic malignancy when up-regulated in EUS-FNAs (*P* < 0.05; Table 3). The Hosmer-Lemeshow test was non-significant (*P* = 0.345) indicating that the model fits the data. Next, the predicted probability of being diagnosed with pancreatic malignancy based on the classifier of two significant predictors was used to construct a ROC curve. This demonstrated that the miR-21 + miR-155 classifier had excellent accuracy for pancreatic malignancy with an AUC of 0.930; and a sensitivity of 81.5% and specificity of 85.7% (Figure 3). Indeed, we also tested all the other possible 2 miRNA combinations (including those not significant at multivariate analysis) and did not find a doublet with a better AUC (Supplementary Table S1). Thus, the combination of miR-21 + miR-155 in EUS-FNAs was able to accurately distinguish between pancreatic carcinoma and benign cases with the best discriminatory power and parsimony.

**Table 3:** Multivariate binary logistic regression of miRNAs as discriminators between malignant and non-malignant pancreatic EUS-FNAs

	B	S.E.	Wald	df	*P*	OR
**miR-21**	0.379	0.168	5.115	1	**0.024**	1.461
**miR-10b**	0.024	0.014	2.954	1	0.086	1.024
**miR-155**	41.737	14.811	7.941	1	**0.005**	1.336 × 10^18^
**miR-196a**	0.044	0.027	2.593	1	0.107	1.045
**miR-210**	−0.080	0.057	1.973	1	0.160	0.923
**miR-135b/24**	0.612	5.141	0.014	1	0.905	1.844

### MiR-21 and miR-155 levels are elevated in pancreatic malignancy in validation cohorts (EUS-FNA and FFPE)

**Figure 4:**
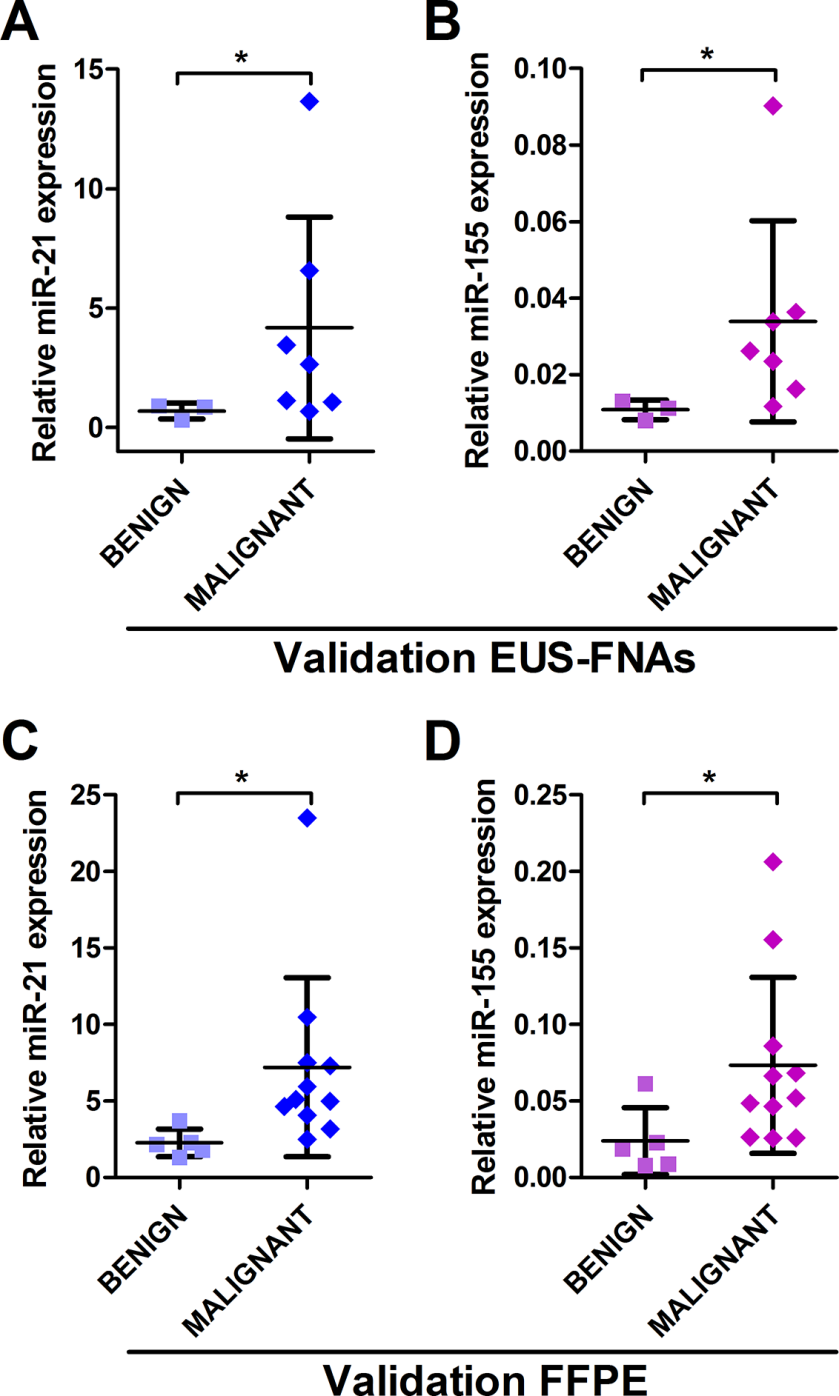
MiR-21 and miR-155 are elevated in pancreatic malignancy in independent EUS-FNA and FFPE cohorts Displayed are the expression levels measured by qRT-PCR across tissue-types for (**A**) miR-21 and (**B**) miR-155 in EUS-FNAs; and (**C**) miR-21 and (**D**) miR-155 in FFPE samples (*P < 0.050).

Next, we validated the 2-miRNA classifier (miR-21 + miR-155) in small independent cohorts of EUS-FNAs (*n* = 10) and archived needle aspirates (FFPE cell blocks; *n = 16*). Clinical details are seen in Supplementary Table S2. Both these miRNAs were significantly up-regulated (*P* < 0.05) in further EUS-FNAs from PDAC lesions compared to benign cases (Figure 4A–4B), as well as in archived FFPE cell blocks from FNAs of invasive IPMNs and PDAC lesions compared to benign cases (Figure 4C–4D).

### MiR-21 and miR-155 levels are elevated pre-procedure in plasma samples from patients with malignant pancreatic lesions

**Figure 5:**
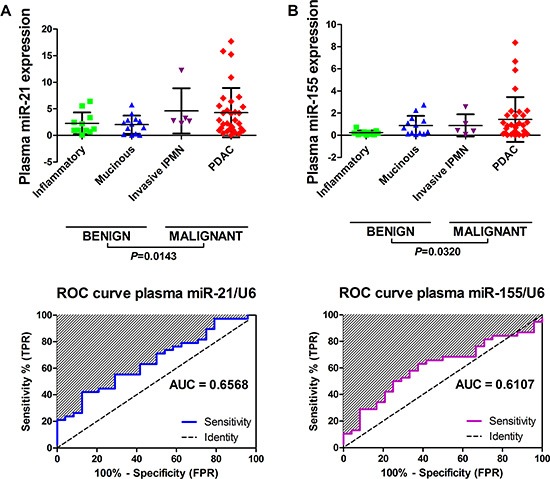
Performance of miR-21 and miR-155 for detecting pancreatic malignancy in plasma samples Displayed are the expression levels across tissue-types and receiver operating characteristic (ROC) curves and area under the curves (AUC) for (**A**) plasma miR-21 and (**B**) plasma miR-155. Scatterplots are shown for each miRNA and the horizontal lines represent the mean expression level and standard deviation. Four groups of pancreatic lesion had plasma samples (*n* = 62) assessed: benign/inflammatory/pseudocysts (*n* = 11); mucinous cysts (*n* = 13); invasive IPMNs (*n* = 5) and PDAC (*n* = 33). One-way analysis of variance (ANOVA) was used to compare plasma miRNA levels between tissue-types, however there was no significant difference between groups. Two-tailed Student's *t*-test was used to compare benign vs. malignant groups.

Next, we analysed pre-procedure plasma samples (*n* = 62) for miR-21 and miR-155 expression. Four groups of patients with pancreatic lesions had plasma samples assessed: benign/inflammatory/pseudocysts (*n* = 11); mucinous cysts (*n* = 13); invasive IPMNs (*n* = 5) and PDAC (*n* = 33). Thirty-four (55%) of these samples were matched with the initial EUS-FNA cohort: benign (*n* = 10); mucinous cysts (*n* = 7); invasive IPMNs (*n* = 2) and PDAC (*n* = 15). We found that neither miR-21, nor miR-155, could distinguish between disease groups, but levels of both were significantly elevated in plasma from patients with malignant compared to benign disease (Figure 5A–5B). However, both plasma miRNAs had poor discriminatory ability for pancreatic malignancy with AUCs < 0.75 (i.e. miR-21, AUC 0.657, 95% CI 0.521–0.793; miR-155 AUC 0.611, 95% CI 0.469–0.753; Figure 5A–5B).

## DISCUSSION

Presently, clinical assessment, focussed imaging and biomarkers (blood, tissue or fluid-based) are unable to fully distinguish benign pancreatic cystic lesions from those at risk of malignant progression [24]. Several studies have measured miRNAs in EUS-FNAs and cystic fluid samples in order to improve the detection of high-risk pancreatic cystic lesions [25]. In this study, we prospectively evaluated 8 miRNAs previously identified as able to detect high-risk or malignant pancreatic lesions in EUS-FNAs.

Our analysis identified a 2-miRNA EUS-FNA classifier, miR-21 + miR-155, which was able to discriminate benign from malignant pancreatic lesions with excellent accuracy (Figure 3). Thus, our study provides further evidence for the potential use of EUS-FNA miRNA markers in assessing pancreatic neoplasms. Importantly, our EUS-FNA 2-miRNA classifier was easily measured in 100% of samples and could discriminate between benign and malignant pancreatic lesions better than standard cytological assessment. This was primarily because cytological evaluation of the same FNAs was hampered by indeterminate results in 36.4% of cases. We were able to validate significant up-regulation of miR-21 and miR-155 in PDAC in a small independent cohort of EUS-FNAs (Figure 4A–4B).

There has been a great interest in miR-21, and miR-155 as biomarkers for pancreatic neoplasia. Habbe *et al.* [18] discovered that miR-21 and miR-155 were up-regulated in *ex vivo* aspirates from surgical specimens of non-invasive IPMNs (*n* = 15), when compared to matched normal pancreata (NP). These miRNAs, however, were unable to discriminate between IPMN adenoma, borderline or carcinoma-in-situ lesions in a larger cohort (*n* = 64) using locked nucleic acid (LNA) *in situ* hybridisation (ISH) techniques [18]. Ryu *et al.* [26] evaluated the expression of five miRNAs (mIR-21, miR-221, miR-17-3p, miR-155 and miR-191) in *ex vivo* pancreatic cyst fluid samples. They found that miR-21, miR-221 and miR-17-3p were significantly up-regulated in the mucinous (*n* = 24) versus the non-mucinous (*n* = 16) cysts [26]. Of these, miR-21 best discriminated between these two types of pancreatic cyst (AUC 0.89; specificity 76% and sensitivity 80%) [26]; however our data could not confirm this (Figure 1A). Consistent with our results, they also found miR-155 was not significantly differentially expressed between the non-mucinous versus mucinous cysts (Figure 1C) [26].

Panarelli *et al.* [21] assessed miRNA expression levels in surgical tissues (PDAC, *n* = 17; IPMNs, *n* = 11; non-neoplastic, *n* = 15) and FFPE cell blocks from FNAs of pancreatic lesions (PDAC, *n* = 35; benign, *n* = 11). They found high levels of miR-21, miR-221, miR-155 and miR-100 in the surgically resected PDACs. However, in the FNAs, high levels of miR-21, miR-221 and miR-196a were able to differentiate PDAC from benign cases. Using a logistic regression model, they created a composite equation using 2 miRNA markers (miR-221 + 2*miR-196a) which had 92% sensitivity and 73% specificity for PDAC. This model also correctly predicted that 89% of cases with equivocal cytological interpretations (i.e. suspicious) were indeed malignant.

Using fresh, pre-operative, endoscopic retrograde cholangiopancreatography (ERCP) pancreatic juice samples, Sadakari and colleagues [22] assessed levels of miR-21 and miR-155 as diagnostic biomarkers. They discovered that levels of both miRNAs were significantly elevated in PDAC samples and able to distinguish these from CP specimens. This finding was also confirmed using matched FFPE tissues [22]. Interestingly, miR-21 and miR-155 expression levels were not correlated with the results of pancreatic juice cytology, however, they were significantly up-regulated in PDAC cases, even when cytological examination found no atypical cells. Indeed, our 2-miRNA classifier of miR-21 + miR-155 was also able to discriminate pancreatic carcinoma from benign cases when standard cytology proved inaccurate.

Other studies have also examined miRNA markers in fresh EUS-FNAs. Szafranska and colleagues identified a 2-miRNA classifier of PDAC (i.e. high miR-196a and low miR-217) in EUS-FNAs [15, 20]. In a subsequent tissue-based study, they discovered a new 2-miRNA classifier for PDAC (i.e. miR-135b/miR-24) that outperformed the first [15]. The miR-135b/24 classifier was able to accurately differentiate PDAC from NP and CP with an AUC of 0.97, and a sensitivity of 92.9% and specificity of 93.4% [15]. In comparison, the miR-196a/217 classifier only had an AUC of 0.78 for detecting PDAC [15]. This study concluded that these assays should be evaluated in conjunction with standard cytology in a large set of EUS-FNAs prospectively collected from patients with benign and malignant pancreatic diseases. Thus, we attempted to validate these 2-miRNA classifiers in our samples. We found that both miR-135b/24 and miR-196a were only moderately predictive at detecting PDAC with AUCs of 0.741 and 0.737 respectively (Figure 2E–2F). We attempted to measure miR-217 expression in our samples, but it was too poorly expressed for accurate assessment, therefore the miR-196a/217 classifier could not be quantified. Another group [21] has also been unsuccessful at measuring miR-217 levels in pancreatic FNAs, thus bringing into question its usefulness. Indeed, Szafranska *et al.* [27] concluded that miR-217 levels are down-regulated in PDAC compared to NP due to a loss of normal acinar cell mass, and therefore may not be the best choice for a normaliser. This group has further validated a 5-miRNA classifier (i.e. miR-135b, miR-24, miR-130, miR-148a and miR-196a) for improving the detection of PDAC in EUS-FNAs [28]. When combined with standard cytology, this 5-miRNA classifier was able to improve the detection of PDAC to 90.8%.

Our previous studies have shown that miR-21 and miR-155 are crucial in pancreatic tumourigenesis. In a tissue-based study [16], we demonstrated that miR-21 and miR-155 levels increase during progression from NP to non-invasive IPMNs and finally to invasive IPMNs. In addition, we found high levels of cyst wall/fluid miR-21 was an independent prognostic marker for patients with CEIs and was associated with shorter overall and disease-free survival. Furthermore, our integrated molecular analysis of PDAC [13] also prioritised miR-21 as a constituent of a triple miRNA combination (miR-21, miR-23a and miR-27a) that appears to drive tumour growth by targeting a network of tumour suppressor genes. Recently, consolidation of miRNA expression profiling efforts has revealed a 10 miRNA meta-signature for PDAC diagnosis (up-regulated: miR-21, miR-23a, miR-31, miR-100, miR-143, miR-155, and miR-221; down-regulated: miR-148a, miR-217 and miR-375), which interestingly includes miR-21 and miR-155 [12, 29]. Our recent meta-analysis [30] of prognostic miRNAs in PDAC revealed that overall-survival was significantly worse in patients with high tissue levels of miR-21 (adjusted HR = 2.48; 1.96–3.14) or miR-155 (adjusted HR = 2.08; 1.26–3.44). Thus, these miRNAs clearly have an important role in PDAC tumourigenesis and prognosis.

Lubezky *et al.* [19] have examined IPMNs of varying degrees of dysplasia, as well as CEI, PDAC and NP samples by microarray. They found that up-regulation of miR-21, miR-155 and miR-708, and a decrease in miR-217 levels occurred during IPMN malignant transformation [19]. Furthermore, Farrell *et al.* [31] found that miR-21 and miR-211 were both able to differentiate benign from malignant pancreatic cysts using EUS-FNAs (*n* = 38). Expression of miR-21 was subsequently validated in matched tissue specimens using ISH and was found to be higher in malignant and pre-malignant cysts compared to benign ones [31]. Finally, in unresectable PDACs, Chung *et al.* [32] have shown that in EUS-FNAs (*n* = 49), high levels of miR-21 were associated with tumour progression and reduced survival compared to low expressors (130 vs. 177 days, *P* = 0.036). Therefore, there is a growing amount of evidence that miR-21 and miR-155 are important in pancreatic tumour progression, and it is unsurprising that we were able to detect pancreatic malignancy using these miRNAs in two cohorts of fresh EUS-FNAs and also archived FFPE cell blocks from FNAs.

Apart from miR-21 and miR-155, we found that miR-10b expression in EUS-FNAs was also able to discriminate between benign and malignant cases with reasonable performance (AUC 0.8307, 95% CI 0.7160–0.9454; Figure 2B). Preis *et al.* [17] demonstrated using ISH on FFPE sections of EUS-FNA samples that miR-10b could differentiate PDAC from NP (PDAC *n* = 95, benign *n* = 11). They went on to find that high miR-10b levels in PDAC EUS-FNAs predicted poor overall and metastasis-free survival, and patients with low miR-10b benefited the most from multimodal neoadjuvant treatment [17, 33]. However, when we entered miR-10b into our multivariate logistic regression analysis, we found that it was not a significant independent discriminator for pancreatic carcinoma (OR 1.024, *P* = 0.086; Table 3) and therefore was not combined with miR-21 and miR-155 for our classifier.

None of the EUS-FNA miRNAs that we evaluated were able to separate benign non-mucinous (i.e. SCAs and inflammatory/pseudocysts) from mucinous pancreatic cysts (i.e. MCN and IPMN), which are crucial to distinguish, as the latter group follow an adenoma-carcinoma sequence. Few studies have been truly successful in this manner. Matthaei *et al.* [14] have used high-throughput miRNA analysis, followed by qRT-PCR in the same FFPE and pancreatic cyst fluid specimens in an attempt to resolve this. Using a novel biomarker discovery approach, they were able to identify differentially expressed pairs of miRNAs (“DiffPairs”) in pancreatic lesions with a “high risk” of malignancy (i.e. high grade IPMNs) and/or those that may require surgery (i.e. solid pseudopapillary neoplasms or pancreatic neuroendocrine tumours). Their logistic regression model, consisted of 9 miRNAs, and correctly separated high risk from low risk pancreatic cysts with a sensitivity of 89%, a specificity of 100% and AUC of 1. Wang *et al.* [24] have gone a step further and used Next-Generation Sequencing (NGS) to examine miRNA expression in EUS-FNAs from low-risk cysts (*n* = 6), high-risk cysts (*n* = 8), and PDACs (*n* = 3). They found 13 miRNAs (miR-138, miR-195, miR-204, miR-216a, miR-217, miR-218, miR-802, miR-155, miR-214, miR-26a, miR-30b, miR-31, and miR-125) were up-regulated and 2 miRNAs (miR-451a and miR-4284) were down-regulated in PDAC. However, these miRNAs were not all significantly differentially expressed after adjusting for false discovery. Interestingly, during qRT-PCR validation, they could not show any significant difference in miR-217 levels in EUS-FNAs from low-risk, high-risk and PDAC lesions. We were unable to detect this miRNA in our samples as it was poorly expressed.

We were also able to assess miR-21 and miR-155 in plasma samples (*n* = 62) taken from patients pre-procedure, of whom 55% (*n* = 34/62) also had EUS-FNA miRNAs measured. In agreement with the recent study by Cote *et al.* [34], we observed increased expression of miRNA-155 in plasma from PDAC patients. However, circulating miR-21 and miR-155 had poor discriminatory ability for identifying malignant disease (Figure 5), compared to EUS-FNA miR-21 and miR-155 (Figure 3).

In summary, there have been few studies to attempt the quantification of miRNAs in fresh EUS-FNAs from pancreatic lesions, and many have focussed on FFPE tissues, as these are easier to obtain. Importantly, studies using surgical FFPE tissues for direct comparison of the miRNA expression with histology have allowed the discovery of PDAC associated miRNAs. There are marked differences in the miRNA profiles from EUS-FNA studies and these variations are undoubtedly due to the different techniques used to collect the samples, isolate total RNA and quantify miRNA levels. Our study is unique in that sample processing started immediately after the FNA was performed as the aspirate was put directly into TRIzol solution and consequently there was no possibility for any contamination, freeze-thaw or time delay, which may result in degradation of the RNA and spurious results.

### Limitations

Limitations of the current study include the preselecting of candidate miRNAs from previously published studies. Many of the studies from which these miRNAs were selected focussed on identifying miRNAs specific for PDAC or IPMNs, and not on differentiating between the other types of pancreatic cystic lesion or distinguishing mucinous from non-mucinous cysts. The next major limitation of our study is the lack of adequate numbers of the different types of IPMN (i.e. epithelial subtypes and also different grades of adenoma). Furthermore, the sample size for each group was small, so we must conclude that non-significant results may be due to Type II error. This is especially true for our inability to reliably detect mucinous cysts. In addition, our analysis only has small independent cohorts of EUS-FNAs and FFPE samples, while lacking larger validation sets, although we stated from the outset that the purpose of the project was to validate findings from previous studies, rather than discover new miRNAs dysregulated in pancreatic lesions. Finally, we were not able to compare our miRNA results with standard clinical biomarkers, such as cyst-fluid CEA levels, as not all patients were tested at the time of echo-endoscopy.

## MATERIALS AND METHODS

### Ethics statement

This study was approved by a London Research Ethics Committee (Camden & Islington 09/H0722/77, 26th November 2009). All patients signed an informed consent form for research prior to an EUS-FNA or blood sampling being performed.

### Study design and patients

**Figure 6:**
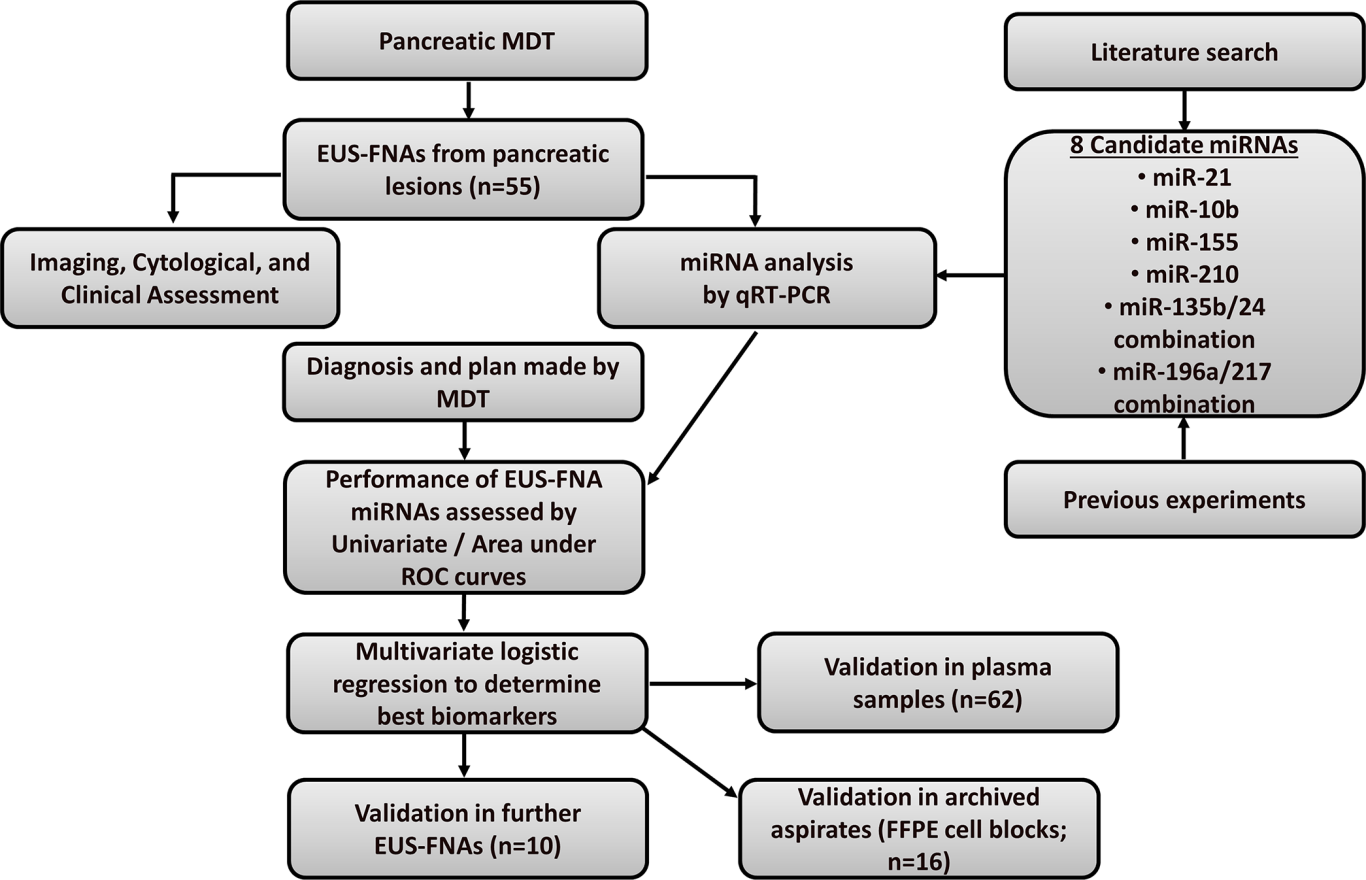
Study design

The initial cohort consisted of patients with a suspicious pancreatic lesion on cross-sectional imaging (CT and/or MRI scan) that were referred for EUS-FNA between 2010–2012 after discussions at the weekly pancreatic multi-disciplinary team (MDT) meeting. Patients were included in the study if they agreed to take part. A flow-chart of the study design can be seen in Figure 6. Patients were followed-up for at least two years and a final diagnosis was determined using histology from surgical resection or from cytology and radiological evidence. Validation was performed on plasma samples (some matched to initial EUS-FNA cohort), and small independent cohorts of EUS-FNAs andarchived biopsies (FFPE cell blocks).

### EUS examination and cytological assessment

Pancreatic lesions were accessed by the transgastric/transduodenal route using a linear echo-endoscope (Pentax Medical, Slough, UK) with a 25 gauge EUS aspiration needle (Boston Scientific, Herts., UK). At echo-endoscopy, patients with lesions suitable for FNA had the first passes sent for cytological assessment. A cytopathology technician was present on site to ensure adequate samples. An additional 1–3 passes were then made and the aspirates placed directly into 250 μl of TRIzol reagent (Life Technologies, Paisley, UK). Samples were then agitated to ensure complete cell lysis and then stored at −80°C until required for RNA isolation. Cytological examination was performed using standard techniques and was always prioritised over miRNA analysis. The FNA material was smeared on microscope slides for on-site examination and/or immediately fixed in 95% ethanol for Papanicolaou staining. The remaining material was placed in a tube containing 4% formaldehyde solution for cell block preparation. Cases were diagnosed according to standard categories as insufficient (C1), negative for malignancy (C2), atypical cells present (C3), suspicious for malignancy (C4) or positive for malignancy (C5). We considered C1, C3 and C4 as inconclusive diagnoses.

### EUS-FNA and FFPE miRNA expression analysis

After careful review of the literature [25], we selected 8 miRNAs for expression analysis in the initial EUS-FNA cohort, including miR-21, miR-155, miR-135b, miR-24, miR-210, miR-196a, miR-217 and miR-10b (Table 4). Four miRNAs have been shown to be 2-miRNA self-normalising classifiers for malignancy (i.e. miR-135b/24 and miR-196a/217) for PDAC in previous studies [15, 20]. RNA was isolated from EUS-FNAs using TRIzol solution according to the manufacturers' protocol. We used TaqMan assays (Life Technologies, Paisley, UK) to measure miRNA expression by quantitative real-time reverse-transcription PCR (qRT-PCR) following the manufacturer's protocol. We used 5ng total RNA for each reverse transcription reaction (RT) for each miRNA for both tissues and plasma. Relative expression was calculated using small nuclear RNA U6 as an endogenous control.

**Table 4:** Candidate miRNAs for detecting pancreatic malignancy in EUS-FNAs

miRNA	Relevance in pancreatic disease	Target genes in PDAC	References
miR-21-5p	Up-regulated in PDAC. Associated with poor prognosis. Enhances resistance to gemcitabine. Up-regulated in non-invasive IPMNs and CEIs.	BTG2, PDCD4, PTEN, SPRY2, RECK, BCL2	[13, 16, 29, 30, 36]
miR-10b-5p	Up-regulated in PDAC. Associated with poor prognosis.	HOXD10, KLF4, TIAM1	[17, 30]
miR-155-5p	Up-regulated in PDAC. Associated with poor prognosis. Up-regulated in non-invasive IPMNs and CEIs.	TP53INP1, FOXP3, PIK3R1	[16, 29, 30]
miR-196a-5p	Up-regulated in PDAC. When normalised with miR-217 (i.e. miR-196a/217), able to detect PDAC with AUC of 0.76 and 0.78.	HOXB8, HMGA2, Annexin A1	[15, 20, 30]
miR-217	Down-regulated in PDAC.	KRAS	[29]
miR-210-5p	Up-regulated in PDAC. Able to detect PDAC with AUC of 0.76. Raised circulating levels in PDAC. Associated with poor prognosis.	EFNA3	[15, 37, 38]
miR-135b-5p	When normalised with miR-24 (i.e. miR-135b/24), able to distinguish PDAC from CP with sensitivity & specificity of 93% and AUC 0.97.	Adenomatous Polyposis Coli in CRC	[15]
miR-24-3p	No specific role in PDAC known. Found to be stably expressed at relatively high levels in all pancreatic cell types. Used for miR-135b/24 classifier.	Member of miR-23a ~ 27a ~ 24–2 cluster and may have overlapping targets	[15]

EUS-FNA, endoscopic ultrasound fine-needle aspiration; IPMN, intraductal papillary mucinous neoplasm; PDAC, pancreatic ductal adenocarcinoma; CEI, carcinoma-ex-IPMN (i.e. invasive IPMN); CP, chronic pancreatitis; CRC, colorectal cancer; AUC, area under the curve.

Further EUS-FNAs and archived needle aspirates (cellular pellets formalin fixed and paraffin embedded into cell blocks) were used for validation of miR-21 and miR-155 by qRT-PCR, as described above. For FFPE samples, RNA was isolated using miRNeasy FFPE Kit (Qiagen, Hilden, Germany) according to the manufacturer's instructions.

### Plasma miRNA expression analysis

Anticoagulated blood (K3-EDTA) was collected from patients prior to EUS-FNA or surgery. Plasma was obtained by standard gradient centrifugation and then immediately frozen at −80°C until required. RNA was isolated from the plasma using the miRNeasy Plasma Kit (Qiagen, Hilden, Germany) according to the manufacturer's instructions. The resulting RNA was evaluated and quantified by spectrophotometry using NanoDrop ND-2000 (ThermoFisher). MiR-21 and miR-155 were measured by qRT-PCR as above. We used small nuclear RNA U6 as an endogenous control [35]. We did evaluate other published endogenous controls, such as miR-16 and miR-425-5p [34], but these exhibited greater inter-sample variability than U6.

### Reference standards for final diagnosis

For patients who underwent surgery, the histological assessment (reference standard) was considered as the final diagnosis. In non-operated patients, the opinion of the MDT (reference standard) was considered as the final diagnosis, including morphological appearance (i.e. EUS, CT and/or MRI), EUS-FNA cytology, biochemical evaluation (i.e. serum CA 19–9), clinical history/examination and follow-up over at least 24 months. The diagnosis was a benign disorder if the clinical course was consistent with investigative findings and if signs of malignancy were absent at the end of follow-up (i.e. disease regression or no evidence of disease progression).

### Statistical analysis

The statistical significance of the continuous variables was tested with a Student's *t*-test, 1-way ANOVA (followed by Tukey's Multiple Comparison tests where appropriate) or Mann–Whitney's *U*-test depending on whether the data were normally distributed. Chi-square test was performed for nominal data. A *P* value < 0.05 was considered statistically significant.

The diagnostic accuracy of miRNA expression at identifying malignancy was compared against the reference standards. The area under the ROC curve (AUC) was used as the measure of a diagnostic test's discriminatory power (i.e. the ability to correctly classify those with or without pancreatic malignancy). We considered an AUC > 0.75 as a clinically useful amount of discrimination (moderate accuracy) and AUC > 0.90 as excellent accuracy.

Multivariate binary logistic regression was then used to select diagnostic miRNA markers based on the initial EUS-FNA dataset. MiRNAs with *P* < 0.05 at univariate analysis (i.e. benign vs. malignant) were entered into the regression model. The predicted probability of being diagnosed with pancreatic malignancy was used to construct a ROC curve, and AUC was used as an accuracy index for evaluating the diagnostic performance of the selected miRNA classifier.

The model performance of the logistic regression analysis was assessed by the Hosmer-Lemeshow test. Summary goodness-of-fit measures describe how well the entire model matches the observed values. A *P* ≥ 0.05 indicated a good logistic regression model.

## CONCLUSIONS

Several studies have found commonly dysregulated miRNAs in PDAC from surgical specimens, biopsies and blood samples. Our study aimed to validate 8 miRNAs previously found to be differentially expressed in pancreatic neoplasia when measured in EUS-FNAs.

Our data show that miRNAs can be quantified easily in EUS-FNAs from pancreatic lesions, and the RNA yield allows the possibility to examine several miRNA species. We identified an EUS-FNA 2-miRNA classifier (miR-21 + miR-155) that was able to distinguish malignant pancreatic lesions from benign ones, with good performance. Most importantly, this 2-miRNA classifier was accurate even when standard cytological assessment of EUS-FNAs was equivocal. Of note, these same miRNAs were not as discriminatory when assessed as possible plasma biomarkers. Nevertheless, our study provides further evidence that miRNAs can enhance standard diagnostic techniques for PDAC.

Continued evaluation miRNAs may lead to a revised miRNA panel with improved sensitivity and specificity for diagnosing PDAC and/or high-risk pancreatic cysts. Larger prospective studies focusing on our candidates and other emerging miRNAs in endoscopically acquired pancreatic FNAs from resectable and non-resectable patients will hopefully allow translation of these molecules into novel biomarkers for clinical use. Further validation may also determine whether miRNA biomarkers in EUS-FNAs can be used to predict patient prognosis and/or guide therapy (e.g. surgical resectability or neoadjuvant / adjuvant chemotherapy).

## SUPPLEMENTARY FIGURES AND TABLES



## References

[1] de Jong K, Nio CY, Hermans JJ, Dijkgraaf MG, Gouma DJ, van Eijck CH, van Heel E, Klass G, Fockens P, Bruno MJ (2010). High prevalence of pancreatic cysts detected by screening magnetic resonance imaging examinations. Clin Gastroenterol Hepatol.

[2] Laffan TA, Horton KM, Klein AP, Berlanstein B, Siegelman SS, Kawamoto S, Johnson PT, Fishman EK, Hruban RH (2008). Prevalence of unsuspected pancreatic cysts on MDCT. AJR Am J Roentgenol.

[3] Jaïs B, Rebours V, Malleo G, Salvia R, Kim M-H, Ha Y, Marchegiani G, Castillo CF-d, Jang J-Y, Kim S-W, Crippa S, Falconi M, Milanetto AC (2014). Pancreatic serous cystadenoma related mortality is almost nil. Pancreatology.

[4] Boot C (2013). A review of pancreatic cyst fluid analysis in the differential diagnosis of pancreatic cyst lesions. Ann Clin Biochem.

[5] Scheiman JM (2008). Management of cystic lesions of the pancreas. J Gastrointest Surg.

[6] Visser BC, Muthusamay VR, Mulvihill SJ, Coakley F (2004). Diagnostic imaging of cystic pancreatic neoplasms. Surgical Oncology.

[7] Hutchins G, Draganov PV (2010). Diagnostic evaluation of pancreatic cystic malignancies. Surg Clin North Am.

[8] Brugge WR, Lewandrowski K, Lee-Lewandrowski E, Centeno BA, Szydlo T, Regan S, del Castillo CF, Warshaw AL (2004). Diagnosis of pancreatic cystic neoplasms: a report of the cooperative pancreatic cyst study. Gastroenterology.

[9] Pitman MB, Lewandrowski K, Shen J, Sahani D, Brugge W, Fernandez-del Castillo C (2010). Pancreatic cysts: preoperative diagnosis and clinical management. Cancer Cytopathol.

[10] Cho CS, Russ AJ, Loeffler AG, Rettammel RJ, Oudheusden G, Winslow ER, Weber SM (2013). Preoperative classification of pancreatic cystic neoplasms: the clinical significance of diagnostic inaccuracy. Ann Surg Oncol.

[11] Cheng Q, Yi B, Wang A, Jiang X (2013). Exploring and exploiting the fundamental role of microRNAs in tumor pathogenesis. Onco Targets Ther.

[12] Frampton AE, Giovannetti E, Jamieson NB, Krell J, Gall TM, Stebbing J, Jiao LR, Castellano L (2014). A microRNA meta-signature for pancreatic ductal adenocarcinoma. Expert Rev Mol Diagn.

[13] Frampton AE, Castellano L, Colombo T, Giovannetti E, Krell J, Jacob J, Pellegrino L, Roca-Alonso L, Funel N, Gall TM, De Giorgio A, Pinho FG, Fulci V (2014). MicroRNAs Cooperatively Inhibit a Network of Tumor Suppressor Genes to Promote Pancreatic Tumor Growth and Progression. Gastroenterology.

[14] Matthaei H, Wylie D, Lloyd MB, Dal Molin M, Kemppainen J, Mayo SC, Wolfgang CL, Schulick RD, Langfield L, Andruss BF, Adai AT, Hruban RH, Szafranska-Schwarzbach AE (2012). miRNA Biomarkers in Cyst Fluid Augment the Diagnosis and Management of Pancreatic Cysts. Clin Cancer Res.

[15] Munding JB, Adai AT, Maghnouj A, Urbanik A, Zollner H, Liffers ST, Chromik AM, Uhl W, Szafranska-Schwarzbach AE, Tannapfel A, Hahn SA (2012). Global microRNA expression profiling of microdissected tissues identifies miR-135b as a novel biomarker for pancreatic ductal adenocarcinoma. Int J Cancer.

[16] Caponi S, Funel N, Frampton AE, Mosca F, Santarpia L, Van der Velde AG, Jiao LR, De Lio N, Falcone A, Kazemier G, Meijer GA, Verheul HM, Vasile E (2013). The good, the bad and the ugly: a tale of miR-101, miR-21 and miR-155 in pancreatic intraductal papillary mucinous neoplasms. Ann Oncol.

[17] Preis M, Gardner TB, Gordon SR, Pipas JM, Mackenzie TA, Klein EE, Longnecker DS, Gutmann EJ, Sempere LF, Korc M (2011). MicroRNA-10b expression correlates with response to neoadjuvant therapy and survival in pancreatic ductal adenocarcinoma. Clin Cancer Res.

[18] Habbe N, Koorstra JB, Mendell JT, Offerhaus GJ, Ryu JK, Feldmann G, Mullendore ME, Goggins MG, Hong SM, Maitra A (2009). MicroRNA miR-155 is a biomarker of early pancreatic neoplasia. Cancer Biol Ther.

[19] Lubezky N, Loewenstein S, Ben-Haim M, Brazowski E, Marmor S, Pasmanik-Chor M, Oron-Karni V, Rechavi G, Klausner JM, Lahat G (2013). MicroRNA expression signatures in intraductal papillary mucinous neoplasm of the pancreas. Surgery.

[20] Szafranska AE, Doleshal M, Edmunds HS, Gordon S, Luttges J, Munding JB, Barth RJ, Gutmann EJ, Suriawinata AA, Marc Pipas J, Tannapfel A, Korc M (2008). Analysis of microRNAs in pancreatic fine-needle aspirates can classify benign and malignant tissues. Clin Chem.

[21] Panarelli NC, Chen YT, Zhou XK, Kitabayashi N, Yantiss RK (2012). MicroRNA expression aids the preoperative diagnosis of pancreatic ductal adenocarcinoma. Pancreas.

[22] Sadakari Y, Ohtsuka T, Ohuchida K, Tsutsumi K, Takahata S, Nakamura M, Mizumoto K, Tanaka M (2010). MicroRNA expression analyses in preoperative pancreatic juice samples of pancreatic ductal adenocarcinoma. JOP.

[23] Hanoun N, Delpu Y, Suriawinata AA, Bournet B, Bureau C, Selves J, Tsongalis GJ, Dufresne M, Buscail L, Cordelier P, Torrisani J (2010). The silencing of microRNA 148a production by DNA hypermethylation is an early event in pancreatic carcinogenesis. Clin Chem.

[24] Wang J, Paris PL, Chen J, Ngo V, Yao H, Frazier ML, Killary AM, Liu CG, Liang H, Mathy C, Bondada S, Kirkwood K, Sen S (2015). Next generation sequencing of pancreatic cyst fluid microRNAs from low grade-benign and high grade-invasive lesions. Cancer Lett.

[25] Frampton AE, Gall TM, Castellano L, Stebbing J, Jiao LR, Krell J (2013). Towards a clinical use of miRNAs in pancreatic cancer biopsies. Expert Rev Mol Diagn.

[26] Ryu JK, Matthaei H, Dal Molin M, Hong SM, Canto MI, Schulick RD, Wolfgang C, Goggins MG, Hruban RH, Cope L, Maitra A (2011). Elevated microRNA miR-21 levels in pancreatic cyst fluid are predictive of mucinous precursor lesions of ductal adenocarcinoma. Pancreatology.

[27] Szafranska AE, Davison TS, John J, Cannon T, Sipos B, Maghnouj A, Labourier E, Hahn SA (2007). MicroRNA expression alterations are linked to tumorigenesis and non-neoplastic processes in pancreatic ductal adenocarcinoma. Oncogene.

[28] Brand RE, Adai AT, Centeno BA, Lee LS, Rateb G, Vignesh S, Menard C, Wiechowska-Kozlowska A, Boldys H, Hartleb M, Sanders MK, Munding JB, Tannapfel A (2014). A microRNA-based test improves endoscopic ultrasound-guided cytologic diagnosis of pancreatic cancer. Clin Gastroenterol Hepatol.

[29] Ma M-Z, Kong X, Weng M-Z, Cheng K, Gong W, Quan Z-W, Peng C-H (2013). Candidate microRNA biomarkers of pancreatic ductal adenocarcinoma: meta-analysis, experimental validation and clinical significance. J Exp Clin Cancer Res.

[30] Frampton AE, Krell J, Jamieson NB, Gall TM, Giovannetti E, Funel N, Mato Prado M, Krell D, Habib NA, Castellano L, Jiao LR, Stebbing J (2015). microRNAs with prognostic significance in pancreatic ductal adenocarcinoma: A meta-analysis. Eur J Cancer.

[31] Farrell JJ, Toste P, Wu N, Li L, Wong J, Malkhassian D, Tran LM, Wu X, Li X, Dawson D, Wu H, Donahue TR (2013). Endoscopically Acquired Pancreatic Cyst Fluid MicroRNA 21 and 221 Are Associated With Invasive Cancer. The Am J Gastroenterol.

[32] Chung KH, Ryu JK, Park JM, Lee JM, Lee SH, Kim Y-T Sa1942 MicroRNA Expression in Unresectable Pancreatic Cancer As a Prognostic Marker in EUS-FNA Cytology Specimens. Gastroenterology.

[33] Frampton AE, Krell J, Jacob J, Stebbing J, Jiao LR, Castellano L (2011). microRNAs as markers of survival and chemoresistance in pancreatic ductal adenocarcinoma. Expert Rev Anticancer Ther.

[34] Cote GA, Gore AJ, McElyea SD, Heathers LE, Xu H, Sherman S, Korc M (2014). A pilot study to develop a diagnostic test for pancreatic ductal adenocarcinoma based on differential expression of select miRNA in plasma and bile. Am J Gastroenterol.

[35] Xu J, Cao Z, Liu W, You L, Zhou L, Wang C, Lou W, Sun B, Miao Y, Liu X, Zhang T, Zhao Y (2015). Plasma miRNAs Effectively Distinguish Patients With Pancreatic Cancer From Controls: A Multicenter Study. Ann Surg.

[36] Giovannetti E, Funel N, Peters GJ, Del Chiaro M, Erozenci LA, Vasile E, Leon LG, Pollina LE, Groen A, Falcone A, Danesi R, Campani D, Verheul HM (2010). MicroRNA-21 in pancreatic cancer: correlation with clinical outcome and pharmacologic aspects underlying its role in the modulation of gemcitabine activity. Cancer Res.

[37] Greither T, Grochola L, Udelnow A, Lautenschlager C, Wurl P, Taubert H (2010). Elevated expression of microRNAs 155, 203, 210 and 222 in pancreatic tumours associates with poorer survival. Int J Cancer.

[38] Ho AS, Huang X, Cao H, Christman-Skieller C, Bennewith K, Le QT, Koong AC (2010). Circulating miR-210 as a Novel Hypoxia Marker in Pancreatic Cancer. Transl Oncol.

